# Differential expression of HIV target cells CCR5 and α4β7 in tissue resident memory CD4 T cells in endocervix during the menstrual cycle of HIV seronegative women

**DOI:** 10.3389/fimmu.2024.1456652

**Published:** 2024-09-25

**Authors:** Sakthivel Govindaraj, Staple Tyree, Gina Bailey Herring, Sadia J. Rahman, Hemalatha Babu, Chris Ibegbu, Marisa R. Young, C. Christina Mehta, Lisa B. Haddad, Alicia K. Smith, Vijayakumar Velu

**Affiliations:** ^1^ Department of Pathology and Laboratory Medicine, Emory Vaccine Center, Emory National Primate Research Center (ENPRC), Emory University, Atlanta, GA, United States; ^2^ Division of Microbiology and Immunology, Emory Vaccine Center, Emory National Primate Research Center, Emory University, Atlanta, GA, United States; ^3^ Department of Gynecology and Obstetrics, Emory University School of Medicine, Atlanta, GA, United States; ^4^ Department of Epidemiology, Rollins School of Public Health, Emory University, Atlanta, GA, United States; ^5^ Grady Ponce de Leon Center, Grady Health System, Atlanta, GA, United States; ^6^ Division of Infectious Diseases, Department of Medicine, Emory University School of Medicine, Atlanta, GA, United States; ^7^ Center for Biomedical Research, Population Council, New York, NY, United States

**Keywords:** HIV target cells, HIV susceptibility, TRM, menstrual cycle phase, endocervix

## Abstract

**Background:**

Ovarian hormones are known to modulate the immune system in the female genital tract (FGT). We sought to define the impact of the menstrual cycle on the mucosal HIV target cell levels, and tissue-resident CD4 T cells.

**Materials and methods:**

Here, we characterized the distribution, phenotype, and function of CD4 T cells with special emphasis on HIV target cells (CCR5+ and α4β7+) as well as tissue-resident memory (TRM; CD69+ and CD103+) CD4 T cells in FGT of cycling women. Peripheral blood and Endocervical cells (EC-collected from cytobrush) were collected from 105 healthy women and performed multicolor flow cytometry to characterize the various subsets of CD4 T cells. Cervicovaginal lavage (CVL) were collected for cytokine analysis and plasma were collected for hormonal analysis. All parameters were compared between follicular and luteal phase of menstrual cycle.

**Results:**

Our findings revealed no significant difference in the blood CD4 T cell subsets between the follicular and luteal phase. However, in EC, the proportion of several cell types was higher in the follicular phase compared to the luteal phase of menstrual cycle, including CCR5+α4β7-cells (p=0.01), CD69+CD103+ TRM (p=0.02), CCR5+CD69+CD103+ TRM (p=0.001) and FoxP3+ CD4 T cells (p=0.0005). In contrast, α4β7+ CCR5- cells were higher in the luteal phase (p=0.0004) compared to the follicular phase. In addition, we also found that hormonal levels (P4/E2 ratio) and cytokines (IL-5 and IL-6) were correlated with CCR5+ CD4 T cells subsets during the follicular phase of the menstrual cycle

**Conclusion:**

Overall, these findings suggest the difference in the expression of CCR5 and α4β7 in TRM CD4 T cell subsets in endocervix of HIV seronegative women between the follicular and luteal phase. Increase in the CCR5+ expression on TRM subsets could increase susceptibility to HIV infection during follicular phase of the menstrual cycle.

## 1 Introduction

The majority of Human Immunodeficiency Virus (HIV) infections in women occur through sexual transmission and across the mucosal barrier (1–3). Although effective antiretroviral therapy (ART) and pre-exposure prophylaxis (PrEP) are available to reduce HIV transmission, acquired immunodeficiency syndrome (AIDS) remains the leading cause of death globally in women (4). Worldwide, women make up almost half of the 1.8 million new cases of HIV infection annually, with most transmissions likely occurring through sexual intercourse (5). Thus, identifying factors associated with increased susceptibility may help target HIV prevention approaches to those at greatest risk.

The Female Genital Tract (FGT) is unique in that it must be immunologically tolerant of a semi-allogeneic conceptus but must also retain the ability to respond to potential pathogens (6). The FGT mucosal immune system consists of epithelial cells, stromal cells, and immune cells which provide a robust physical and immunological barrier against sexually transmitted infections (STIs) (7, 8). The leukocytes dispersed throughout FGT tissues comprise 6-20% of total cells, the majority of which are T-lymphocytes (9). A study also suggests that FGT might also be a port of entry for HIV-1 following sexual intercourse (10), where HIV-susceptible target cells CCR5+, α4β7+ CD4 T cells are abundant (11). Although the mechanisms of HIV transmission in the FGT are an active area of investigation, sex-based differences in immune responsiveness, genetic factors, and the effects of sex hormones may alter individual susceptibility (12). Hormonal fluctuations during the menstrual cycle may enhance vulnerability to HIV infection via altering the expression of HIV receptors and co-receptors by CD4 T cells (13). An earlier study also extended this in SHIV-infected pigtail macaques demonstrating periodic shifts in the immune response under menstrual cycle regulation drive bystander CCR5+ CD4 T cells in the female reproductive tract (14, 15). Studies have suggested that the localization of HIV target cells in the mucosal tissues is an indicator of susceptibility to HIV infection in humans (16). HIV acquisition appears to correlate with the frequency of activation-associated properties and CCR5 expression on CD4 T cells in the blood (17, 18) as well as expression of integrin α4β7 (19). Thus, conditions that increase CCR5, α4β7, and immune activation in both circulating and tissue-resident CD4 T cells identify increased HIV infection risk (19).

Menstruation includes elements that are consistent with an inflammatory response, even when no infection is present (20). Earlier studies have proposed that during menstrual cycle regulation, immune suppression occurring during greater ovarian progesterone production increases HIV susceptibility in the FGT in humans (16, 21, 22) and susceptibility to SHIV in the FGT of pigtail macaques (15, 23). Moreover, the preclinical and clinical study model shows that the phases of the menstrual cycle has been previously linked with increased susceptibility to Chlamydia trachomatis, Candida albicans, and Neisseria gonorrhoeae infections (24–27). However, progesterone has an inhibitory effect on HIV infectivity, and *ex-vivo* FGT cross-sectional infection studies provide conflicting evidence as to whether HIV replication is more likely to occur at either the follicular or luteal phase (28, 29). These findings underscore critical gaps in our understanding of how the menstrual cycle can impact HIV target cells and the key mechanisms that drive HIV risk from sexual exposure.

To evaluate HIV susceptibility across the menstrual cycle, we used samples from healthy women to study immune properties with HIV risk factors over time. We studied the phenotype of fresh CD4 T cells isolated from the EC and peripheral blood, collected from healthy HIV seronegative women during the different phases of the menstrual cycle. CD4 T cells from EC had a predominantly activated phenotype with increased expression of CCR5, α4β7, CD69, and CD103 compared to blood. Notably, follicular phase endocervical CD4 T cells from the EC significantly enriched for CCR5+, CD69+, CD103+, Ki67+, and FoxP3+ cells, as compared to the luteal phase of the menstrual cycle, suggesting high susceptibility to HIV-1 infection. In addition, we found that plasma hormonal levels, the P4/E2 ratio were associated higher levels of CCR5+ CD4 T cells during the follicular phase. Furthermore, we analysed cytokines in EC, we found that IL-2 and MCP-1 were higher in follicular phase compared to luteal phase and the Th2 cytokines (IL-5 and IL-6) were associated with CCR5+ CD4 T cells subsets during the follicular phase of the menstrual cycle. These results reveal that endocervical CD4 T cells have a phenotype that may render them highly susceptible to infection by HIV-1 and other sexually transmitted pathogens when progesterone production is decreased. Although some data suggest progesterone levels may increase HIV risk (30, 31), data from earlier studies echo our findings that progesterone has an inhibitory effect on HIV infectivity (28, 29). Overall, our results demonstrate menstrual cycle regulation of the CD4 T cell activation and tissue-resident CD4 T cells.

These data suggest that P4/E2 ratio is associated higher levels of CCR5 during the follicular phase, and the cytokines IL-5 and IL-6 are associated with CCR5+ CD4 T cells subsets during the follicular phase of the menstrual cycle.

## 2 Materials and methods

### 2.1 Study design and participants

We recruited HIV seronegative women with an intact uterus and cervix; all were between 18–44 years old. They participated in visits during the follicular and luteal phases as previously described (32). Subjects were recruited from several sites in the greater Atlanta community including referrals from clinical sites and other studies, via posters and social media, and web-based recruitment through the National Research Match project (https://www.researchmatch.org). Negative HIV serostatus was confirmed at the time of screening using an FDA-approved HIV antibody test. This protocol was approved by the Institutional Review Board at Emory University and the Grady Research Oversight Committee. Written informed consent was obtained from all participants.

### 2.2 Study inclusion and exclusion criteria

Females between 18-45 years of age were eligible to participate if they (1) had normal menses (occurring within 22-35 day intervals) for at least two cycles; (2) had an intact uterus and cervix; (3) were interested in initiating HC and willing to accept DMPA, ENG-implant, or LNG-IUD; (4) willing to delay initiation of HC for up to one month; (5) able and willing to provide informed consent and undergo study procedures; (6) had a negative HIV test by OraQuick^®^ method at the screening visit; (7) and agreed to abstain from vaginal intercourse and abstain from use of intra-vaginal products for one day prior to each study visit.

Women were not eligible for the study if they met any of these criteria (1): pregnant or planning to become pregnant within the next year; (2) breastfeeding, if not having regular, active menstrual cycles; (3) history of cervical loop electrosurgical excision procedure (LEEP), conization, or cryosurgery within the past year; (4) current use of systemic HC or IUD, based on self-report and/or hormonal testing; (5) concurrent medications that interact with selected HC; (6) contraindications to selected contraceptive per the Centers for Disease Control (CDC) and Prevention Medical Eligibility Criteria or judgment of the study clinician.

### 2.3 Specimen collection

Paired blood (B), and endocervical cells (EC) collected using cytobrush (CB) were obtained during the follicular (between 7–10 days from the start of the previous menstrual bleed) and luteal (between 21–25 days from the start of the previous menstrual bleed) phases during consecutive visits. Sexual, reproductive, medication and genitourinary symptom histories were collected at each study visit. Cervical specimens were collected by the trained study clinician for all participants during speculum pelvic examination; genital specimens were collected according to strict protocol as previously described (32). Two sequential endocervical cell samples were collected using two cytobrushes which were immediately stored in 20 ml vials of complete media (RPMI -1640, penicillin, streptomycin, L-glutamine with 10% human serum). Following collection, specimens were immediately transported on ice for real-time processing and immune phenotyping. The presence of visible blood contamination was assessed in all FGT specimens at the time of sample collection, and blood contamination was removed using FACS lysis buffer at the time of processing the endocervical cell samples; cervicovaginal lavage was tested for semen using the ABACard^®^ p30 antigen detection test. Serological tests for HIV and HSV are performed via enzyme immunoassay (EIA) using a Vitros^®^ 3600 Immunodiagnostic system (Ortho Clinical Diagnostics) (32).

### 2.4 Sample processing

All samples were processed within three hours of collection. CBs were processed at 4˚C as described by McKinnon et al. (33). CBs were inserted into a 25 ml serological pipette containing 15 ml complete media. The CB was moved in and out of the pipette tip to dislodge the cells, while the CB was washed in complete media to extract cells into a 50ml conical tube. Cells were then strained through a 100μm strainer once. Endocervical cell suspensions were washed once with 10ml complete medium and resuspended in 100μl of complete medium and used for flow cytometric staining. After the determination of cell counts, cells were used immediately for flow cytometry. The peripheral blood mononuclear cells (PBMCs) were isolated from whole blood collected in sodium citrate tubes and isolated by density gradient centrifugation according to standard procedures as described previously (32, 34).

### 2.5 Flow cytometry

PBMC was isolated from peripheral blood, and endocervical cells collected from CB were stained in PBS containing 2% FBS for 30 min at room temperature (RT). Cells were stained with fluorochrome-conjugated antibodies specific for, CD3 (SP34-2), CD4 (L200), CD8 (RPA-T8), CCR5 (3A9), HLADR (SK1), Ki67 (B56) from BD Biosciences (San Jose, CA); CD45 (HI30), CD56 (HCD56), CD38 (HIT2), CD69 (FN50), FoxP3 (206D) from Biolegend, (San Diego, CA); CD103 (HML-1) from Beckman Coulter (Brea, CA); and α4β7 (ACT-1) from the NIH Nonhuman Primate Reagent Resource. Dead cells were excluded from the analysis based on staining for Live/Dead NearIR dead cell stain from Molecular Probes, Invitrogen. Staining for FoxP3 was performed after cells were stained for surface antigens followed by permeabilization/fixation using the FoxP3 kit and protocol, followed by intracellular staining. Samples were acquired on an LSR Fortessa (BD Biosciences) and all cellular events were collected for mucosal samples while 500,000 events were collected for samples from blood.

### 2.6 Hormone assessment

We stratified the menstrual cycle phase (follicular and luteal) using plasma hormone levels and measured the values of estradiol (E2), progesterone (P4), P4/E2 ratio, follicular-stimulating hormone (FSH), and luteinizing hormone (LH) values using ultra-high-performance liquid chromatography–heated electrospray ionization-tandem triple quadrupole mass spectrometry (LC-MS/MS) on a Shimadzu Nexera-LCMS-8050 instrument (Kyoto, Japan) and used LabSolutions Software, V5.72 (Shimadzu) for all the processing and analysis performed by Endocrine Technologies Core at the Oregon National Primate Research Center (35, 36).

### 2.7 Cervicovaginal lavage samples processing and measurement of vaginal cytokine concentration by MesoScale

CVL specimens are tested for prostate-specific antigen (PSA), a marker for semen exposure, using the Abacus ABAcard^®^ p30 test (Abacus Diagnostics, West Hill, CA). Testing for CVL blood and leukocyte levels is also performed with Mutistix^®^ 8SG urinalysis strips (Siemens Healthcare, Los Angeles, CA). CVL samples are centrifuged to separate into supernatant and cellular fractions; 8 mL of the CVL supernatant is collected, and aliquots are labeled and stored at -80°C for subsequent assays as previously described (32). CVL samples were tested for measurement of cytokines/chemokines (IFN-γ, TNF-α interleukin (IL)-1α, IL-1β, IL-2, IL-4, IL-5, IL-6, IL-8, IL-10, IL-12p70, IL-17α, IL-18, MCP-1 (CCL2), MIP-1α, MIP-β, Eotaxin (CCL11), and IP-10) using the MesoScale assay platform (Meso Scale Diagnostics Rockville, Maryland), which uses electrochemiluminescence for high sensitivity and broad dynamic range, according to the protocols supplied by the manufacturer. Samples were run in duplicate and averaged, and duplicates with a coefficient of variation >15% were not analyzed further.

### 2.8 Data and statistical analysis

Menstrual cycle phases were classified based on hormone levels: luteal (P4 ≥1000 pg at visit 1 or ≥3000 pg at visit 2) and follicular (<1000 pg at visit 1 or <3000 pg at visit 2). Data were analyzed using FlowJo software v X.10.0 after gating out dead cells. Participant clinical and demographic characteristics are described by count and percentage for categorical characteristics and mean and standard deviation for continuous characteristics. Within each specimen (EC, blood), T cell subset frequencies and frequency of CD4 TRM markers (HIV co-receptors, activation markers, and mucosal trafficking markers) were assessed for association with cycle phase (follicular, luteal). To incorporate the appropriate repeated measures within a participant (visit, cycle phase), mixed effect models were used and included a random intercept for participants with an unstructured covariance matrix adjusting for age (years) and race (Asian, Black/African American, White/Caucasian, Else). There was no difference in cellular markers by race, and thus race was not included as a covariate in any models. To investigate tissue-resident memory cell expression of CCR5 and α4β7, the models described above were stratified by co-expression of CD69 and CD103. Due to low relative frequency, for CD69 and CD103, generalized mixed effect gamma models with a logit link were used. Within blood only, separate models assessed each hormone level (E2, LH, FSH) with cycle phase (luteal, follicular). Model-based mean estimates and 95% confidence intervals are reported. Model fit was assessed by residual plots. Co-expression of α4β7 with CCR5 and co-expression of CD69 with CD103 were modeled as percentages of total cells of each co-expression marker combination (PosPos, NegPos, PosNeg, NegNeg). Independent variables in each model included marker combination and cycle phase, all possible interaction terms, and included age and race as covariates. Both co-expression models included a random intercept for the subject and robust variance estimation and were stratified by specimen type. The correlation was assessed using Spearman correlation at the first visit if a participant had two visits with the same cycle phase. Statistical analyses were performed in SAS 9.4, plots were created in GraphPad Prism. Analyses are exploratory with α=0.05.

## 3 Results

### 3.1 Participants demographic/study design

**Figure 1:**
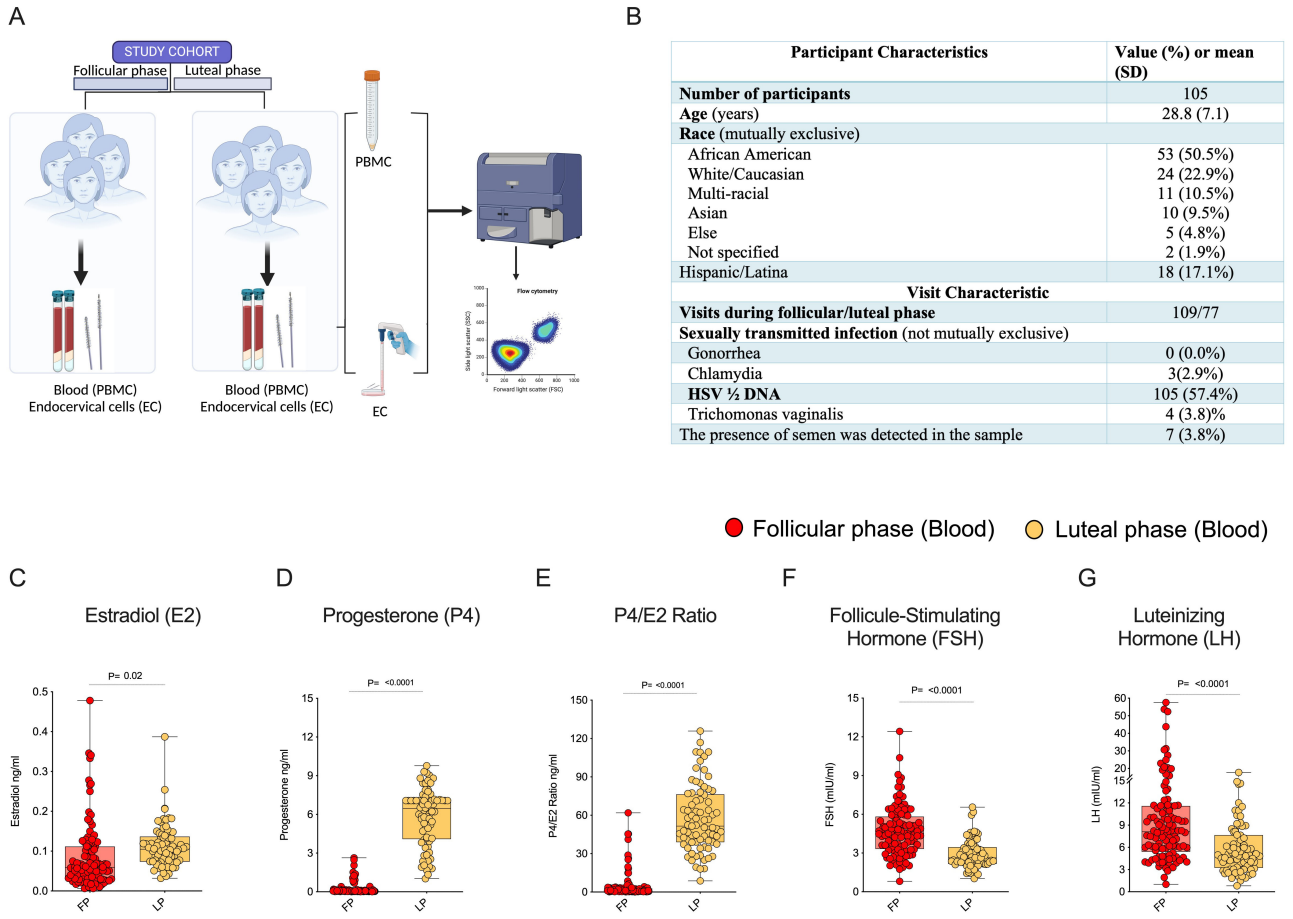
Immune cells expression in Female Genital Tract (FGT): **(A)** Healthy adult females were recruited for the study during the luteal and follicular phase of the menstrual cycle, cells were isolated from endocervical samples and blood samples to study the expression of immune markers in the FGT & systemic compartments. **(B)** Clinical and demographic characteristics of participants (column percent may not total 100 due to rounding). Blood plasma measurements of female reproductive hormone profiles **(C)** Estradiol (E2), **(D)** Progesterone (P4), **(E)** P4/E2 Ratio, **(F)** Follicle-Stimulating Hormone (FSH), **(G)** Luteinizing Hormone (LH).

This analysis included 105 HIV seronegative women with multicolor flow cytometry immune profiling and hormonal assays to confirm their menstrual cycle phase (**Figure 1A**). Participants were non-contraceptive users recruited in Atlanta, Georgia to participate in a study evaluating the impact of contraceptive use on immunologic factors in the female genital tract. The data in this evaluation is from the initial 2 visits prior to initiation of their choice of contraceptive. Demographic, clinical, and visit characteristics of the participants are shown in (**Figure 1B**). The mean participant age was 28.8 years, 50% of participants were Black/African American, 23% were white, and 17% were Hispanic/Latina. The majority of participants had not recently engaged (within the last 3-5 days) in condomless sex, as supported by the ABAcard^®^ p30 test for the forensic identification of semen. A few Chlamydial and Trichomonas infections were seen among the participants during visit recruitment (**Figure 1B**). All women in the study were monitored for hormonal levels in plasma. The luteal phase is characterized by higher levels of E2, P4, and P4/E2 ratios, and the follicular phase is characterized by elevated levels of the luteinizing hormone (LH) (**Figures 1C–G**). Next, we analyzed cellular immune markers (CD45+ leukocytes, CD3+ T cells, CD4+ T cells) from specimens (Blood and EC) and compared the expression of CD4+ HIV target cells (CCR5, α4β7), activation (CD38, HLADR), proliferation (Ki-67), tissue-resident memory markers (CD69, CD103), and T regulatory marker (Fox-P3) between the follicular and luteal phases. The data revealed that there were no significant variations in the blood between the follicular and luteal phases. However, we did find differences in EC, thus below we primarily focus on immune marker expression in EC.

### 3.2 CD4 Target cells are higher in the endocervix during the follicular phase compared to the luteal phase of the menstrual cycle

**Figure 2:**
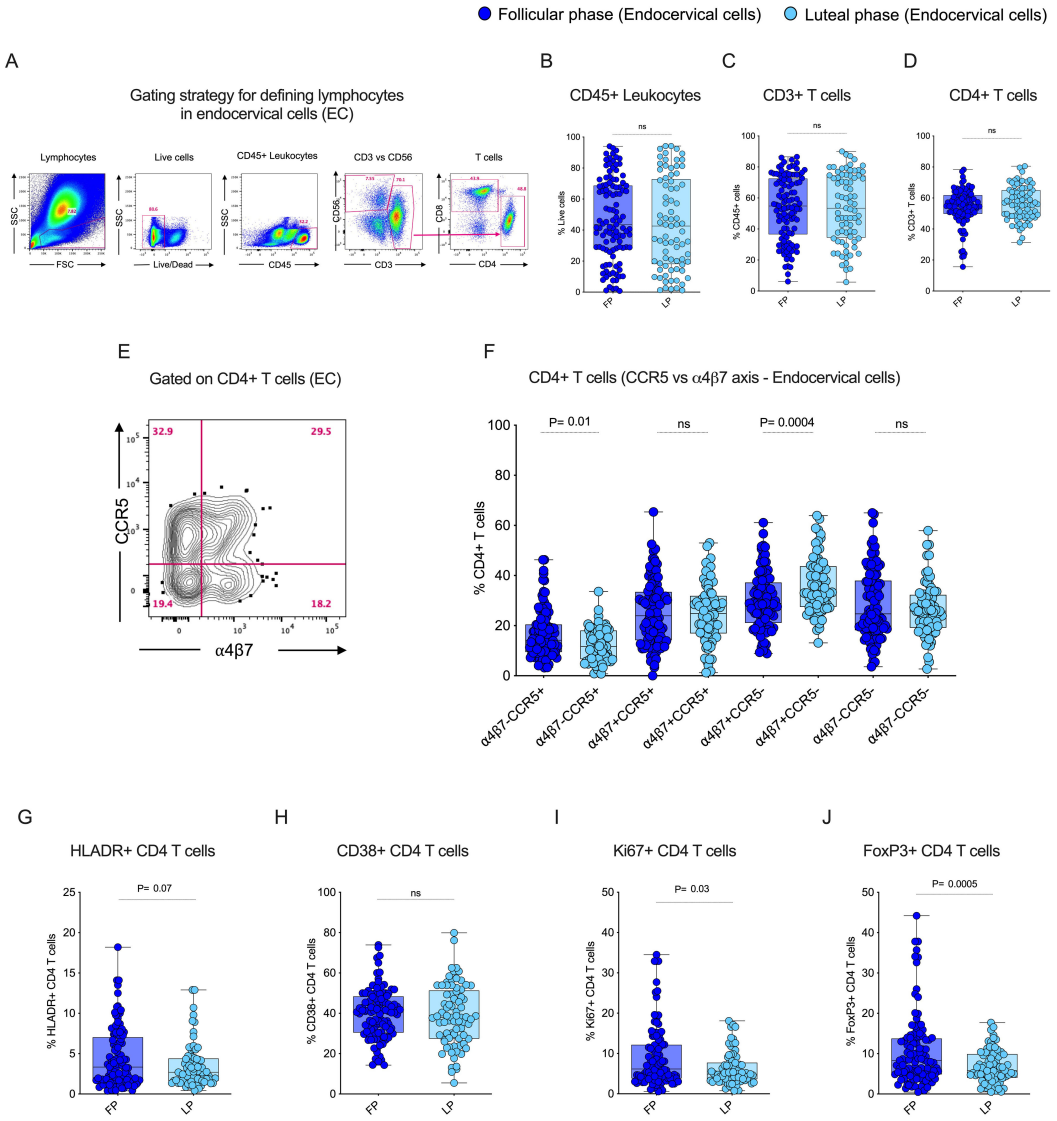
Follicular phase of the menstrual cycle expresses elevated levels of HIV target cells, proliferation, and regulatory T cells in the Female Genital Tract (FGT): **(A)** Gating strategy to identify T cells from endocervical cell (EC) samples. **(B)** Box plots showing the frequency of CD45+ leukocytes. **(C)** Frequency of CD3+ T cells. **(D)** Frequency of CD4+ T cells during the follicular phase (FP) and luteal phase (LP) of the menstrual cycle in EC. **(E)** Gating pattern for HIV target cells. **(F)** CD4+ α4β7 and CCR5 co-expression in EC. **(G)** Frequency of HLA-DR+ CD4 T cells in EC. **(H)** Frequency of CD38+ CD4 T cells in EC **(I)** Frequency of Ki67+ CD4 T cells in EC. **(J)** Frequency of FoxP3+ CD4 T cells in EC during the follicular phase (FP) and luteal phase (LP) of the menstrual cycle.

To understand the influence of the menstrual cycle’s follicular and luteal phase on CD4 T cells in blood and endocervix, we used flow cytometry to identify CD4+ T cells based on the live cells, CD45+ leukocytes for analysis. A representative flow plot of the gating strategy used to identify CD4 T cells in endocervical samples is shown in **Figure 2A**. No significant difference was observed in the total leukocytes, CD3+ T cells, and CD4 T cells (**Figures 2B–D**) between the two phases of the menstrual cycle within each specimen type, which is consistent with previously reported data (34). The average value of cellular markers within each cycle phase and the specimen is given in **Supplementary Table 1**. We then compared the HIV target cell markers CCR5 and α4β7 within the EC between the follicular and luteal phases. The gating pattern of target cells CCR5 and α4β7 mono and co-expression in EC is shown in **Figure 2E**. The total CCR5+ CD4 T cells were significantly higher (p=0.02) in the follicular phase compared to the luteal phase of EC (**Supplementary Table 1**). We then analyzed the co-expression pattern of target cells with respect to CCR5 and α4β7. Similar to the total CCR5+ CD4 T cells, the target cells expressing α4ß7-CCR5+ CD4 T cells were significantly elevated (p=0.01) in the follicular phase in the EC samples (**Figure 2F**; **Supplementary Table 2**). In contrast, the HIV binding protein expressing α4ß7+CCR5- CD4 T cells was significantly elevated (p=0.0004) in the EC samples from the luteal phase compared to the follicular phase (**Figure 2F**). Yet, there is no difference observed in α4ß7 vs CCR5 CD4 T cells in the blood between the follicular and the luteal phase (**Supplementary Figures 1A–F**, **Supplementary Tables 3**, **4**). These data show that within EC, the follicular phase expresses a higher level of CCR5, whereas the α4ß7 expression is significantly higher in the luteal phase, suggesting there is a differential expression of target cell markers between the follicular and luteal phases of the menstrual cycle.

### 3.3 CD4 T cell proliferation and regulatory T cell expression are higher in the endocervix during the follicular phase of the menstrual cycle

The CD4 T cell expression of activation markers HLADR and CD38, proliferation marker Ki67, and regulatory marker FoxP3 were analyzed in the endocervix during the follicular and luteal phase of the menstrual cycle. There was no difference in activation observed between the follicular and luteal phases in the EC for either HLADR or CD38 expression (**Figures 2G, H**). However, there was a difference in the expression of Ki-67 (p=0.03) and FoxP3 (p=0.0005), which were significantly higher in EC during the follicular phase compared to the luteal phase (**Figures 2I, J**). These markers were unchanged in the blood between the follicular and the luteal phase (**Supplementary Figures 1G–J**, **Supplementary Table 3**). These findings demonstrated that, although there was no significant activation, expression of proliferation and regulatory markers differ by phase in the endocervix.

### 3.4 Enhanced expression of tissue-resident memory CD4 T cells in the endocervix during the follicular phase compared to the luteal phase of the menstrual cycle

**Figure 3:**
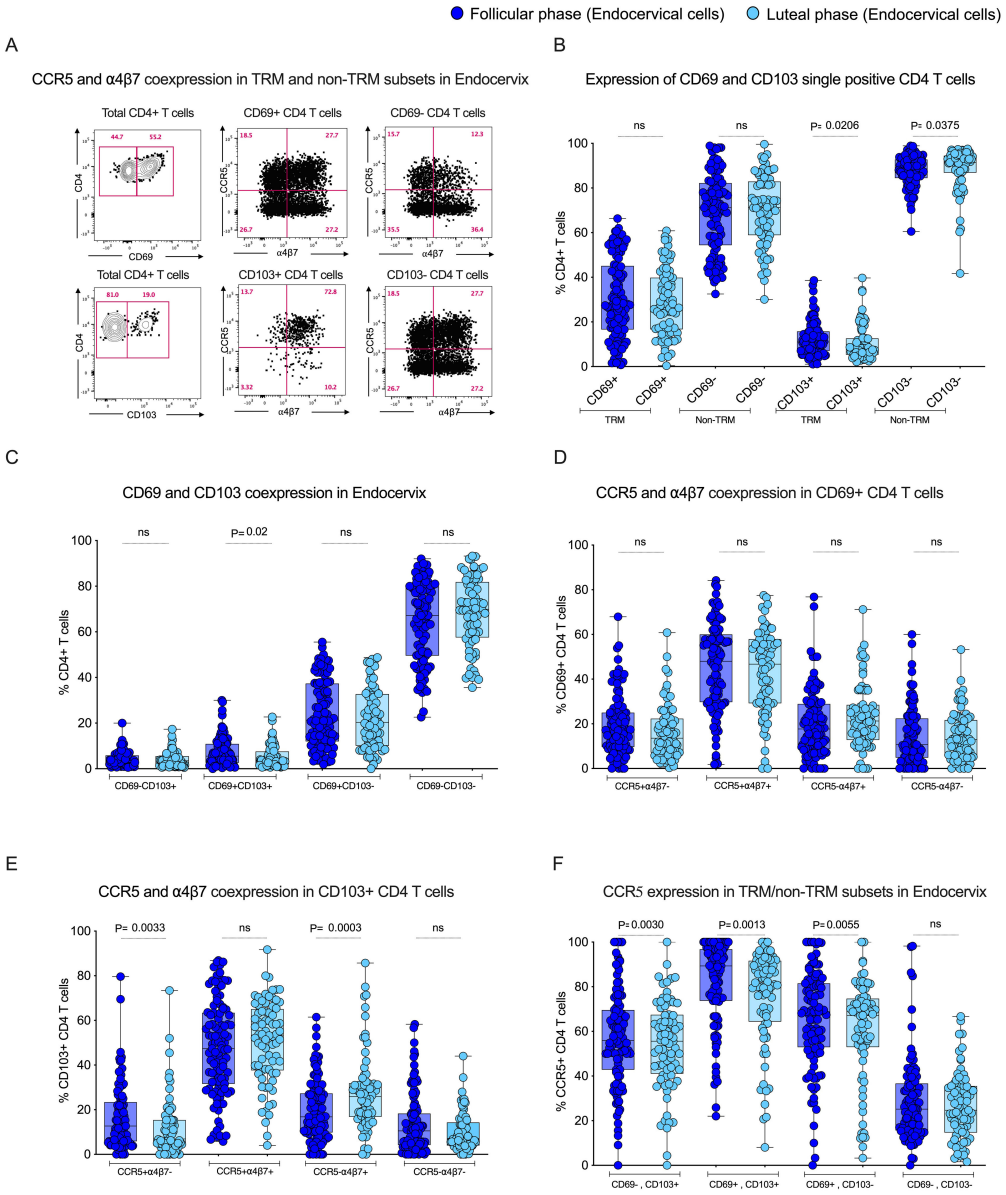
Tissue-resident subsets express higher levels of HIV target cells CCR5 during the follicular phase of the menstrual in the Female Genital Tract (FGT): **(A)** Representative plot for CCR5 and α4β7 co-expression in TRM and non-TRM subsets in Endocervix. **(B)** Box plot depicts expression of CD69 and CD103 single positive cells in EC. **(C)** CD69 and CD103 co-expression in EC. **(D)** CCR5 and α4β7 co-expression in CD69+ CD4 T cells in the FP and LP. **(E)** CCR5 and α4β7 co-expression in CD103+ CD4 T cells in the FP and LP. **(F)** Expression of CCR5 in tissue-resident subsets in endocervical cells (EC) during the follicular phase (FP) and luteal phase (LP) of the menstrual cycle.

To study the influence of tissue-resident memory CD4 T cells in the endocervix, we utilized flow cytometry to identify tissue-resident CD4 T cells based on key markers for tissue residency CD69 and CD103. CD69 serves as a retention signal and inhibits the expression of sphingosine-1-phosphate receptor 1 (S1PR1), a receptor required for T cell egress from tissues to lymphatics, thereby retaining memory T cells in tissue without circulation. CD103 promotes the retention and localization of T cells within tissue epithelial barriers by binding to E-cadherin. We compared the tissue-resident markers CD69, CD103 single positive and CD69+CD103+ double-positive expression between the two different phases of the menstrual cycle in EC. First we anlaysed the data individually with respect to frequency of total TRM markers CD69+ and CD103+ CD4 T cell population. We observed that CD69+ CD4 T cells are dominant in EC compared to the CD103+ CD4 T cells. Although there is no difference in the total CD69+ CD4 T cells between the follicular and luteal phase in EC and blood, the frequency of total CD103+ CD4 T cells were higher in the follicular phase both in EC and blood (**Figures 3A, B**; **Supplementary Figure 2A**). Then we analyzed the TRM with respect to quadrant gate within the CD69 and CD103 axis. The boolean analysis showed the co-expression CD69+CD103+ CD4 T cells (p=0.02) were higher during the follicular phase compared to the luteal phase in the endocervix but not in the blood (**Figure 3C**), these values are similar in the blood between the follicular and the luteal phase (**Supplementary Figure 2B**). These data demonstrate that the CD69+ cells are higher in EC with higher CD69+CD103+ CD4 T cells in the follicular phase of EC.

### 3.5 The tissue-resident memory CD4 T cells express higher levels of CCR5 in the endocervix during the follicular phase of the menstrual cycle

To understand the influence of the menstrual cycle phase on HIV target cell expression within the tissue-resident compartment of the endocervix, we measured the expression of CCR5+, α4β7+ and CCR5+α4β7+ co-expression within the tissue-resident memory CD4 T cell subsets. First, we analyzed the expression of both CCR5 and α4β7 within the total CD69+ and CD103+CD4 T cells. The total TRM cells, CD69+ and CD103+ CD4 T cells express higher levels of CCR5+ cells compare to non-TRM cells. Interestingly, the non-TRM cells express higher levels of α4β7 in EC in both of the phases (**Supplementary Figures 2C–F**). We also see similar trends in blood-derived CD69+ and CD103+ CD4 T cells (**Supplementary Figures 3A–D**). We then compared the CCR5 and α4β7 expression within the total CD4 T cells between the follicular and luteal phases. We found no difference in the single or co-expression profile of CCR5 and α4β7 within the total CD69+ CD4 T cells (**Figure 3D**). However the expression of CCR5+ single positive (CCR5+α4β7-) cells were higher in the follicular phase, and the expression of α4β7+ single positive (CCR5-α4β7+) cells was higher in the luteal phase within the CD103+ CD4 T cells (**Figure 3E**). We then analyzed the CCR5 and α4β7 data with TRM cells with respect to quadrant gate within the CD69 and CD103 axis. The data revealed that the CCR5 expression was higher during the follicular phase of the menstrual cycle in the TRM subsets, including CD69-CD103+ (p=0.003), CD69+CD103+ (p=0.001), CD69+CD103- (p=0.005), when compared to luteal phase in EC (**Figure 3F**). Conversely to the CCR5 expression, there was a significant difference observed in α4β7 expression in the EC within the CD103+ TRM CD4 T cell subset (**Supplementary Figures 3E–G**). In contrast to the EC, the blood CCR5+α4β7+ cells were higher CD69+ CD4 T cells in the luteal phase and CCR5-α4β7+ cells were higher in the CD103+ CD4 T cells in the follicular phase, and no difference in the expression of CCR5 and α4β7 in non-TRM cells between the phase. (**Supplementary Figures 4A–F**). These data suggest that the TRM subsets in the endocervix may be more susceptible to HIV infection during the follicular phase due to their higher expression of HIV co-receptor CCR5.

### 3.6 Target cells CCR5, α4β7 and regulatory cells Fox-P3 are positively associated with tissue-resident memory CD4 T cell subsets in EC

**Figure 4:**
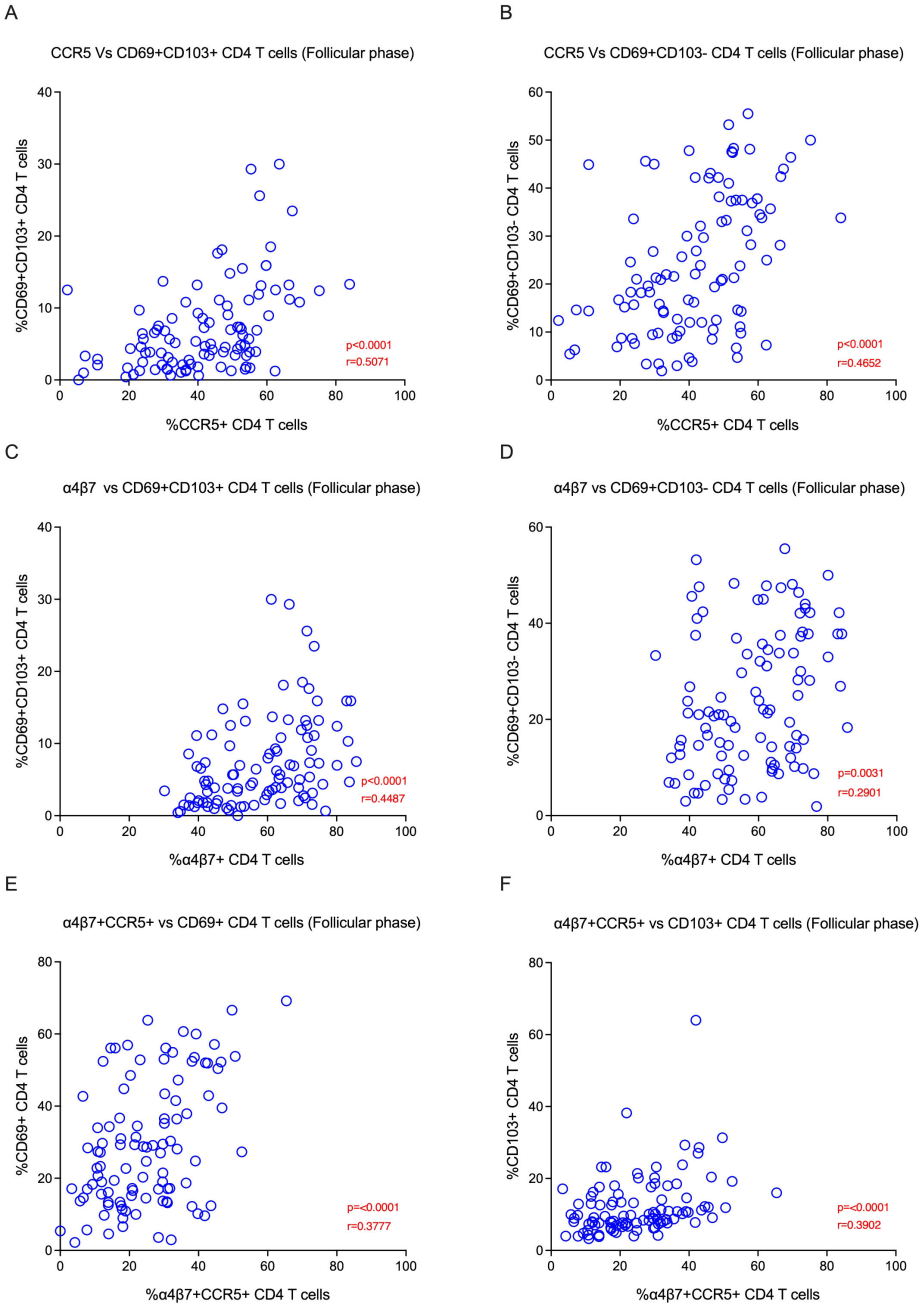
HIV target cells are positively correlated with tissue-resident memory subsets in the Female Genital Tract (FGT) during the follicular phase: **(A)** The plot represents the Spearman correlation between CCR5 vs CD69+CD103+ CD4 T cells **(B)** Correlation between CCR5 vs CD69+CD103- CD4 T cells. **(C)** Correlation between α4β7 vs CD69+CD103+ CD4 T cells. **(D)** Correlation between α4β7 vs CD69+CD103- CD4 T cells. **(E)** Correlation between α4β7+CCR5+ cells vs CD69+ CD4 T cells. **(F)** Correlation between α4β7+CCR5+ cells vs CD103+ CD4 T cells in the endocervical cells (EC) during the follicular phase (FP) of the menstrual cycle.

**Figure 5:**
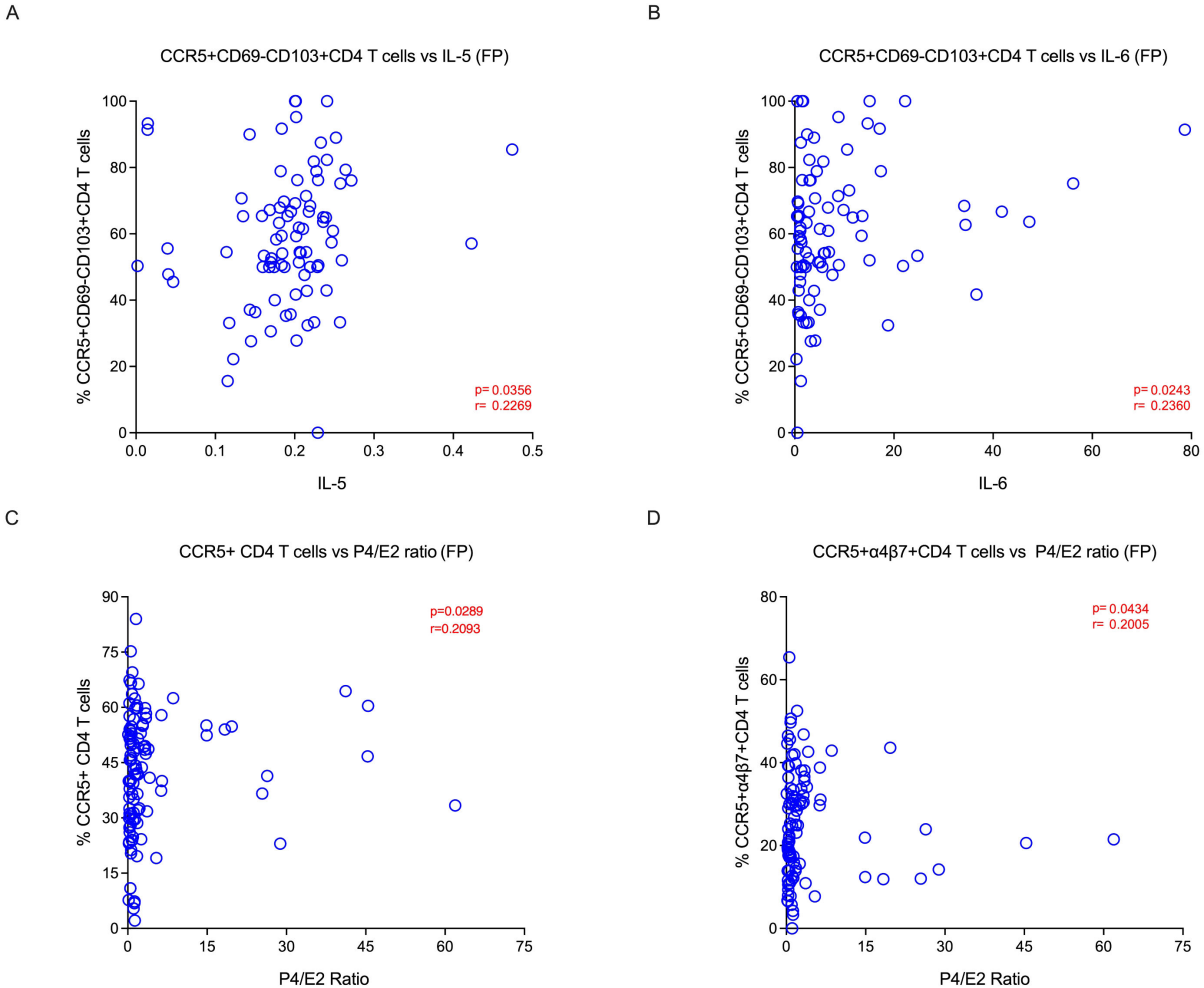
Tissue-resident target cells are positively correlated with P4/E2 hormonal ratio and Th2 cytokines during follicular phase: **(A)** Correlation between IL-5 vs CD69-CD103+CCR5+ CD4 T cells and **(B)** IL-6 vs CD69-CD103+CCR5+ CD4 T cells during the follicular phase. **(C)** Correlation between P4/E2 ratio vs target cells CCR5+CD4 T cells and **(D)** CCR5+α4β7+CD4 T cells during the follicular phase of menstrual cycle in the endocervical cells (EC).

We sought to elucidate the relationship between CCR5, α4β7 and Fox-P3 expression, and tissue-resident memory markers. We evaluated various TRM subsets and their correlations during different phases of the menstrual cycle in the EC. We found that HIV co-receptor CCR5 expression was positively associated with endocervix TRM subsets CD69+CD103+ (p<0.0001) and CD69+CD103- (p<0.0001) during the follicular phase (**Figures 4A, B**). In addition, the α4β7 expression was positively associated with endocervical TRM subsets CD69+CD103+ (p<0.0001) and CD69+CD103- (p=0.003) during the follicular phase (**Figures 4C, D**). Similarly, HIV target cells CCR5 vs. CD103+ T cells (p<0.001) and α4β7 vs CD103+ CD4 T cells (p=0.01), α4β7 vs. CD69+CD103+ CD4 T cells (p=0.04) are positively correlated with tissue-resident markers in the luteal phase of the menstrual cycle in EC (**Supplementary Figures 5D–F**). We also examined the association of target cell subsets within the α4β7 vs CCR5 quadrant with TRM subsets. The α4β7+CCR5+ CD4 T cells were associated with both CD69+ and CD103+ TRM subsets (**Figures 4E, F**; **Supplementary Tables 5A, B**). We then examined the association of CCR5+α4β7+ double positive cells with TRM cells, similar to the single positive cells the double positive cells correlated with the both CD69 and CD103 TRM subsets (**Figures 4E, F**). We then asked whether there is an association between target cells and cytokines in the EC. We found that Th2 cytokines IL-5 and IL-6 correlated positively CCR5+ TRM subsets in follicular phase (**Figures 5A, B**) and cytokine MIP-1β correlated with the TRM cells that express α4β7 in luteal phase (**Supplementary Figure 5G**). Changes in the proportion of regulatory CD4 T cells have been reported during the menstrual cycle (37). However, its association with TRM subsets is not understood. Hence, we examined the correlation between the Fox-P3 expression and TRM subsets during the different phases of the menstrual cycle. We found positive correlation between tissue-resident markers and the FoxP3+ CD4 T cells; FoxP3 vs. CD69+CD103- CD4 T cells (p=0.02), FoxP3 vs. CD69-CD103+ CD4 T cells (p=0.0006), FoxP3 vs. CD69+CD103+ CD4 T cells (p=0.001) in the follicular phase (**Supplementary Figures 5A–C**) but not in the luteal phase. These data suggest that both target cells and regulatory T cells are associated with TRM subsets during the follicular phase of the menstrual cycle.

### 3.7 Hormonal ratio (P4/E2) and Th2 cytokines were associated with higher levels of CCR5+ CD4 T cells in the follicular phase of mensural cycle.

Ovarian hormones have been shown to modify the immune and inflammatory response (21). Hence, we sought to elucidate the relationship between hormonal levels and the cellular markers between the two phases. We looked at various CD4 T cells, including TRM and non-TRM subsets, and their association with different hormonal levels within the follicular and luteal phases of the menstrual cycle in the EC. We found that CCR5 expressing cells, such as CD4+CCR5+, CD4+α4β7+CCR5+, and CD4+CD69+CD103-CCR5+ cells, were associated with P4/E2 ratio during the follicular phase (**Figures 5C, D**; **Supplementary Table 6A**). However, markers pertaining to activation (CD4+CD38+ and CD4+Ki-67+ cells) are associated with E2, P4 and P4/E2 and are higher in the luteal phase. The CD4+α4β7-CCR5+ and CD4+CD69+CD103+ cells were associated with higher levels of FSH. In blood, we did not identify any target cells that associated with hormone levels, however the activated CD4+CD69+ cells correlated with both P4 and P4/E2 ratio (**Supplementary Tables 6A, B**). We then compared the level of various cytokines in the endocervix between the follicular and luteal phases (**Supplementary Figure 6**). We found that IL-2 and MCP-1 levels were higher in the follicular phase. These two cytokines are important for proliferation and migration of T cells (38). In addition, we found that these two cytokines were associated with E2 and LH levels in the follicular phase (**Supplementary Table 6C**). Then we sought to elucidate the relationship between cytokine levels and the cellular markers in the two phases. We examined various CD4 T cell subsets and their association with different cytokine levels within the follicular and luteal phases of the menstrual cycle. We found that Th2 cytokines, IL-5 and IL-6, were associated with higher levels of CD4+CD69-CD103+CCR5+ cells in the follicular phase. However the CD4+α4β7+CCR5+ and CD4+CD69+CD103+α4β7+ cells were associated with IL-17 and MIP-1β in the luteal phase (**Supplementary Tables 7A, B**). These data suggest that P4/E2 ratio is associated higher levels of CCR5 during the follicular phase, and the cytokines IL-5 and IL-6 are associated with CCR5+ CD4 T cells subsets during the follicular phase of the menstrual cycle.

## 4 Discussion

**Figure 6:**
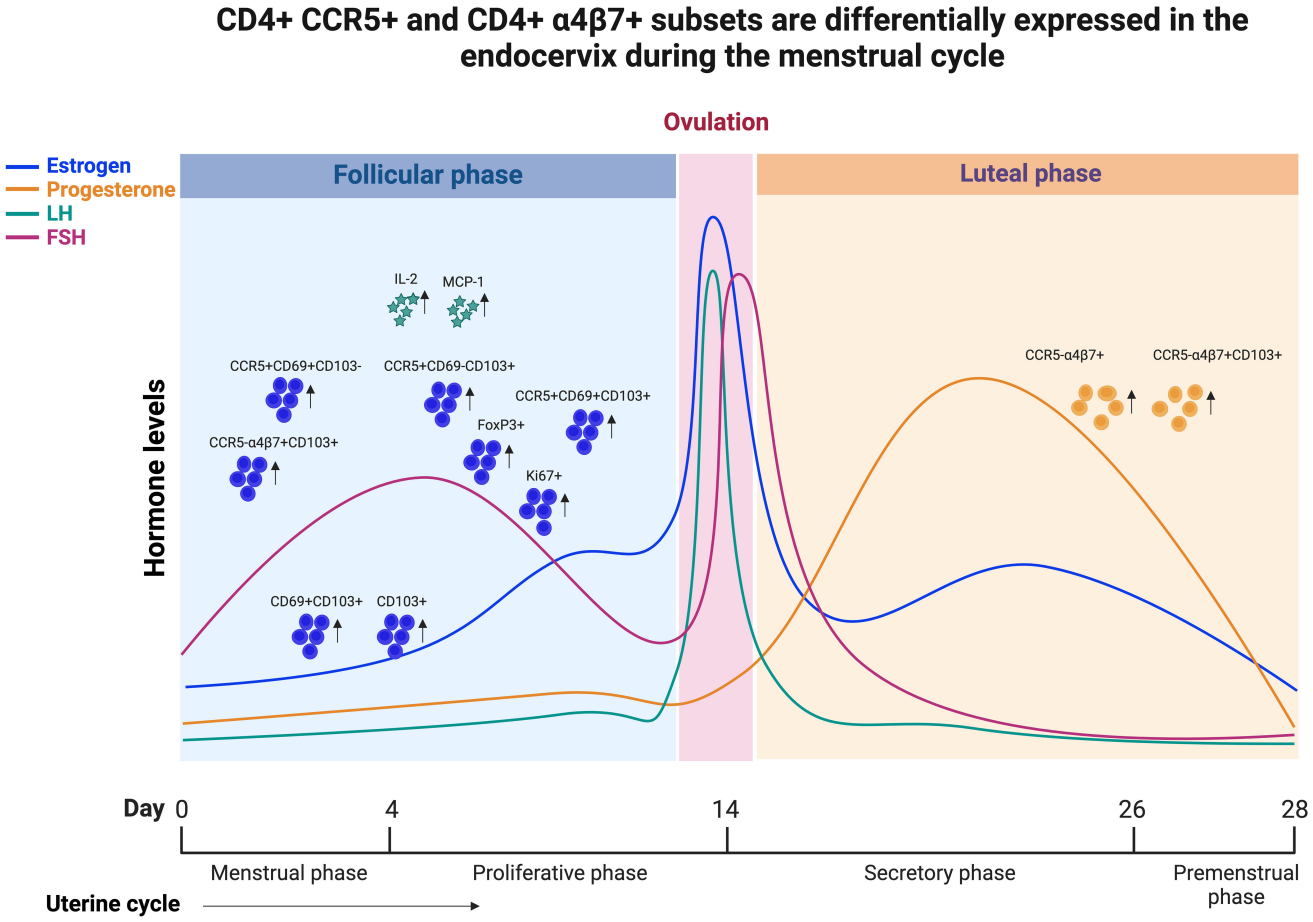
Graphical abstract demonstrating that CCR5+ and α4β7+ CD4 T cells are differentially expressed in the endocervix during menstrual cycle of HIV seronegative women.

Female sex hormones are known to regulate the innate and adaptive immune system (39). However, the fluctuation of these hormonal levels and their influence on the tissue-resident CD4 T cells that express CCR5 and α4ß7 during the menstrual cycle phases is not clearly understood (40). This study aimed to investigate the influence of the menstrual cycle phase on immune cell activation, with special emphasis on the immune markers related to HIV susceptibility. We characterized the CD4 T cell markers pertaining to HIV target cells, HIV binding protein, activation, proliferation, and tissue residency in EC and compared their expression between the luteal and follicular phases of the menstrual cycle. First we found that CCR5 expression on CD4 T cells are elevated during the follicular phase. In addition, we found increased expression of tissue-resident markers with higher expression of CD69+CD103+ double-positive cells in the follicular phase of EC compared to the luteal phase. We also found that the CCR5 expression is higher in TRM compared to non-TRM subsets, but the non-TRM cells express higher levels of α4β7. Of note, this CD69+CD103+ double-positive population expresses significantly higher levels of CCR5 in the follicular phase compared to the luteal phase in EC, summary of our results are depicted as graphical abstract in **Figure 6**.

Although there was no overall difference in activation of total CD4 T cells between follicular and luteal phases, the CCR5+ TRMs activation were higher in the follicular than the luteal phase, suggesting a very specific increase in susceptibility in these TRMs, and highlighting that CCR5+ TRMs could be more susceptible to HIV infection during the follicular phase of the menstrual cycle. Our data is consistent with an earlier study identifying CD4+ TRMs as primary targets for HIV infection, viral reservoir, and viral persistence, and the female genital tract as an HIV sanctuary (10). These data suggest that enrichment of HIV target cells during the follicular phase of the menstrual cycle makes FGT vulnerable to higher infection risk following sexual exposure. Our findings support an experimental study involving non-human primates, which indicates that Simian human immunodeficiency virus (SHIV) infection was observed in the follicular phase of 88% of macaques, with only 12% of macaques getting infected during the luteal phase with repeated vaginal virus exposures (15). Our results confirm and align with earlier data indicating that HIV co-receptor CCR5+ CD4 T cells were more abundant during the follicular phase of the menstrual cycle in endocervical cells (21), and it is known that low CCR5 expression protects HIV-specific CD4 T cells of HIV elite controllers from viral entry (18). The current findings echo earlier study suggesting an elevated level of CCL2 and retention of resident memory CD4 T cells during the follicular phase which could make them more susceptible to infection compared to the luteal phase (21). In addition, our findings support α4β7 expression increases during the luteal phase of the menstrual cycle in EC. This is consistent with prior data indicating that elevated frequency of α4β7+ CD4 T enhanced infection in the vaginal explants model (41), and blocking α4β7 protects macaques from vaginal SHIV acquisition (41, 42). Apart from facilitating HIV binding and entry (19, 43), α4β7 directs migration of CD4 T cells to the gut and could traffic HIV-infected cells from the FGT to sites of active replication in the gut, thereby facilitating the establishment of HIV infection (44, 45). A better understanding of the phenotype and viral permissivity of CCR5, α4β7 co-expression on target cells in the FGT should be evaluated in future human studies.

Our data demonstrate increased tissue-resident markers, the double positive (CD69+CD103+) CD4 T cells during the follicular phase in EC. The expression of proliferating CD4+ T cells and tissue-resident memory CD4 T cells in EC were higher during the follicular phase of the menstrual cycle. This result is consistent with a recent clinical study performed among sex workers in Kenya, which demonstrated the higher expression of CD69 on CD4+ resident T cells during the follicular phase. The same study also observed an elevated proportion of CCR5+CD69+ CD4 T cells in FGT during the follicular phase (21), which we confirmed, demonstrating, that the tissue-resident CD69+CD103+ subset expresses higher levels of CCR5 in EC during the follicular phase. A study evaluating a non-human primate model of HIV infection in pigtail macaque also suggests that the late luteal phase is associated with infiltration of CCR5+ CD4 T cells into the vaginal lumen (40). Furthermore, expression of CD69 demonstrates recent activation and is highly consistent with the phenotype of tissue effector memory (TEM) cells, which are highly enriched in the mucosa and non-lymphoid immune sites (46, 47). These cells rapidly produce effector cytokines upon antigen exposure (48, 49) with mouse studies showing their indispensable role in protection against HSV2 and other mucosal infections (50, 51). Vaginal TEMs represent highly vulnerable HIV target cells due to their activated state and the expression of CCR5, and studies in macaques strongly implicate the role of vaginal TEMs as founder cells contributing to the local viral expansion and viral dissemination (11, 52, 53). We see differences in the expression of CCR5 and α4β7 between phases of the menstrual cycle. We observed that the TRM mostly express CCR5 compared to α4β7, and the non-TRM mostly express α4β7. These differences could be due to their expression pattern in various CD4 T cell subsets pertaining to memory compartment. In general the CCR5 is expressed in memory CD4 T cells compared to naïve CD4 T cells. On the other hand the α4β7 is expressed in both memory and naïve CD4 T cell compartment. We observe direct association of target cells with TRMs, hormonal ratio (P4/E2) and cytokines IL-5, IL-6 and MIP-1β in the follicular phase. At this time, it is not clear why higher levels of CCR5+ CD4 T cells were observed in the follicular phase. We hypothesize that these changes could be driven by various factors such as hormones, cytokines, microenvironment and microbiome.

We demonstrated the presence of T reg cells in the FGT with increased expression during the follicular phase. This result is also consistent with the earlier studies, where the elevation of FOX-P3+ CD4 T cells is noted in the follicular compared to the luteal and late luteal phase (40, 54), although our study did not differentiate between early and late luteal phase. The frequency of CD4 T regs in blood has previously been shown to correlate with estradiol levels, peaking during the late follicular phase of the menstrual cycle when estrogen levels are the highest (54), and while some findings suggest that estrogen induces FoxP3 expression *in vitro* (55), other findings demonstrate this *in vitro* and *in vivo* (56), thereby supporting their potential contribution to hormonally mediated changes in HIV susceptibility. Although the present study was not designed to capture dynamic changes in T reg frequencies within the FGT in the presence of varying endogenous or exogenous reproductive hormones, the ability to use longitudinal FGT sampling methods is critical to evaluating changes in these cells in the presence of reproductive hormones in future studies.

This study has limitations. First, our generalizability may be limited as this was a single-site study conducted in the United States among sexually active women. However, the high enrollment of African American women, who are disproportionately impacted by HIV, as well as the similar results among other study (34) enhance the confidence of the results beyond this cohort. Another limitation is that the small sample size with little variability of multiple covariates such as vaginal infections, the presence of semen and endogenous or exogenous hormones, which can impact HIV risk, in part, by altering the phenotype of FGT CD4 T cells, that may affect the study’s outcome.

## 5 Conclusion

To conclude, our study provides valuable new insights into the target cell availability with tissue resident phenotype in FGT across the menstrual cycle. We anticipate that novel HIV prevention strategies that reduce infiltration of target cells into the FGT may help reduce HIV acquisition and transmission in women.

## Data Availability

The original contributions presented in the study are included in the article/**Supplementary Material**. Further inquiries can be directed to the corresponding authors.
