# Supplementary material for: Differential expression of HIV target cells CCR5 and α4β7 in tissue resident memory CD4 T cells in endocervix during the menstrual cycle of HIV seronegative women
Source: Front Immunol. 2024 Sep 25;15:1456652. doi: 10.3389/fimmu.2024.1456652 (PMC11461385; doi:10.3389/fimmu.2024.1456652)
Supplement: Supplementary file 1 [file Table1.docx]

**Supplementary Materials**

**Supplemental Figures:**

**
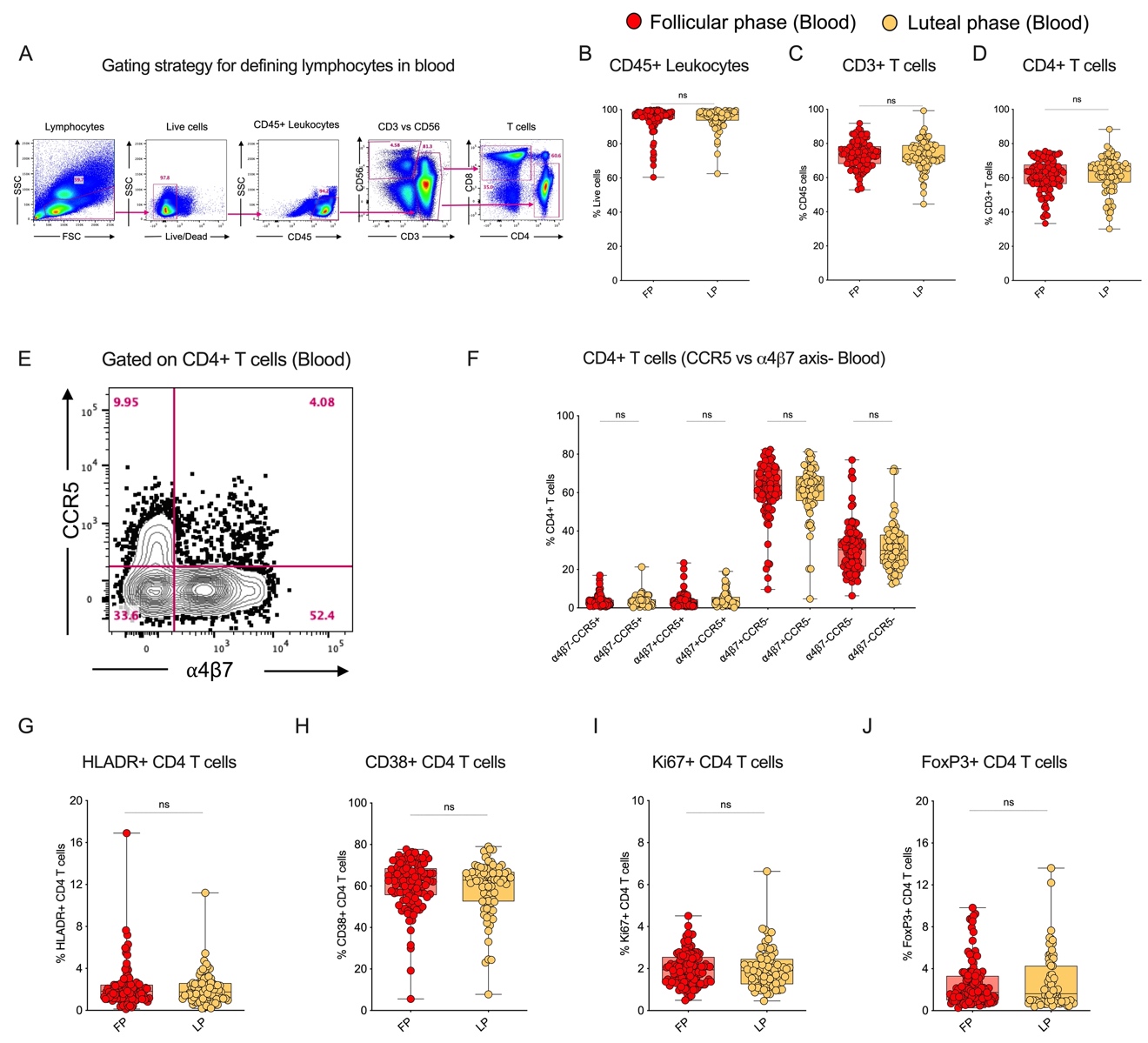
**

**Supplemental Figure 1. The frequency of CD4 T cells, HIV target cells, activation, proliferation, and regulatory markers are unchanged in the blood during the follicular and luteal phase of the menstrual cycle:** **(A)** Gating strategy to identify T cells in the blood. **(B)** The Box plot represents the frequency of CD45+ Leukocytes. **(C)** Frequency of CD3+ T cells. **(D)** Frequency of CD4+ T cells. **(E)** Representative plot for gatting of HIV target cells in the blood. (**F)** CD4+ α4β7 vs CCR5 expression in blood during the follicular phase (FP) and luteal phase (LP) of the menstrual cycle. **(G)** Frequency of HLA-DR+ CD4 T cells. **(H)** Frequency of CD38+ CD4 T cells. **(I)** Frequency of Ki67+ CD4 T cells **(J)** Frequency of FoxP3+ CD4 T cells in blood during the follicular phase (FP) and luteal phase (LP) of the menstrual cycle.


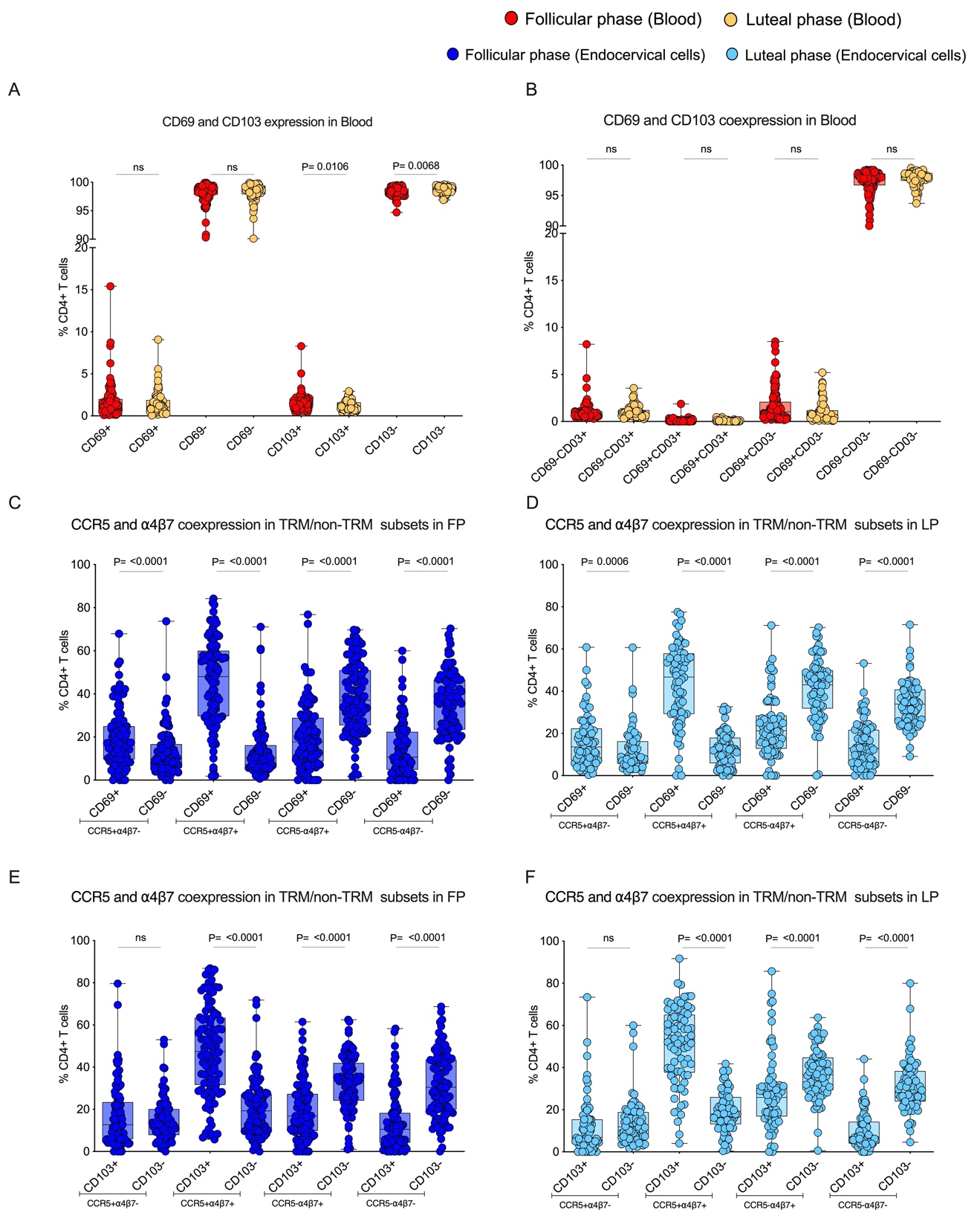


**Supplemental Figure 2. The expression of CCR5+α4β7-, CCR5+α4β7+ cells expressed higher in TRM and CCR5-α4β7+, CCR5-α4β7- were higher in non-TRM subsets during the follicular and luteal phase of the menstrual cycle: (A)** Box plot shows CD69 and CD103 single positive cells in the blood. **(B)** CD69 and CD103 coexpression in the blood. **(C)** CCR5 and ⍺4β7 coexpression in CD69+ & CD69-ve subests in the FP of EC. **(D)** CCR5 and ⍺4β7 coexpression in CD69+ & CD69-ve subests in the LP of EC. **(E)** CCR5 and ⍺4β7 coexpression in CD103+ & CD103-ve subests in the FP of EC. **(F)** CCR5 and ⍺4β7 coexpression in CD103+ & CD103-ve subests in the LP of EC

**
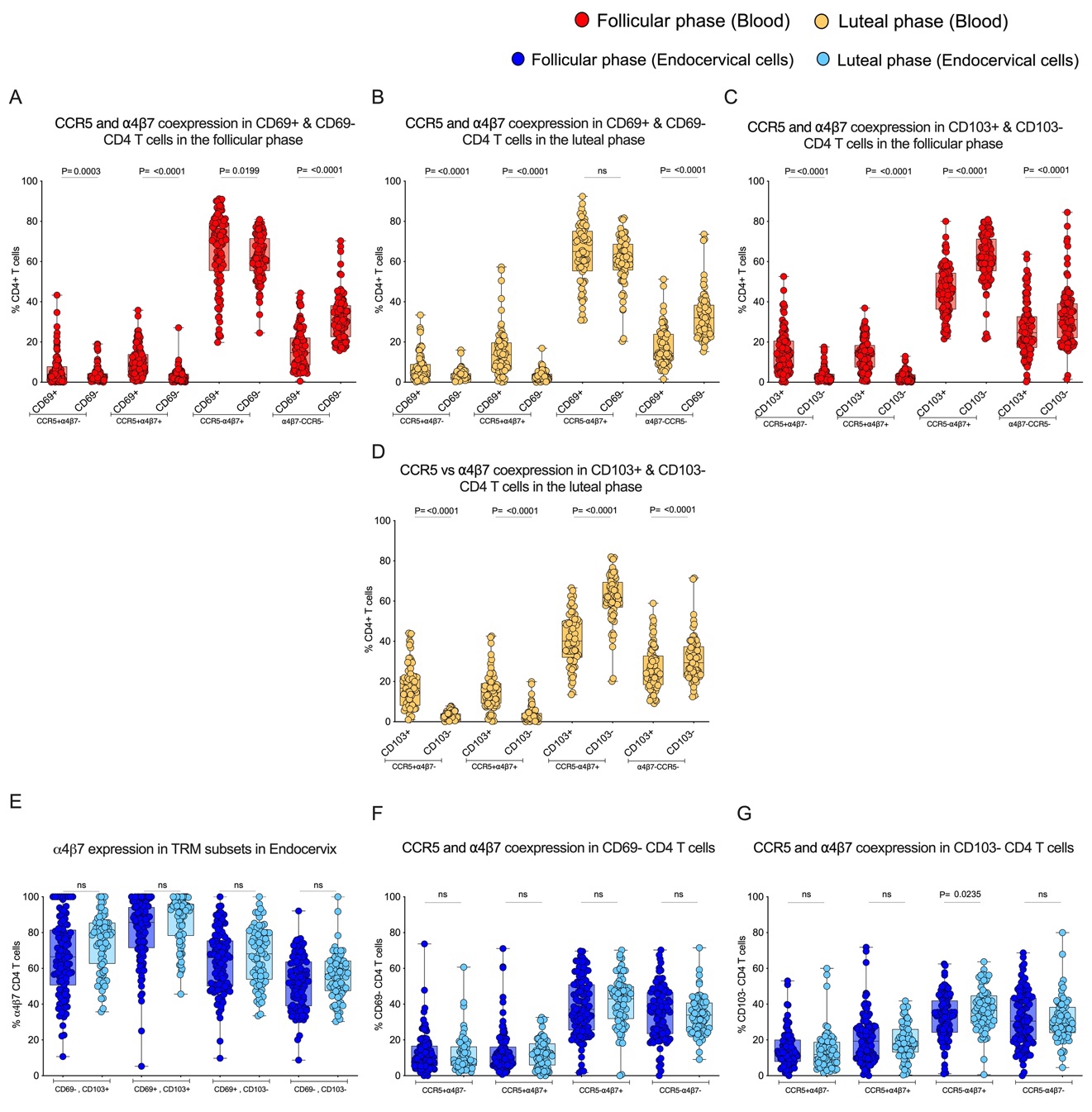
**

**Supplemental Figure 3. The expression of α4β7 and CCR5 in the CD69 vs CD103 axis is similar in the blood between the follicular and luteal phase of the menstrual cycle: (A)** The box plot represents the coexpression of CCR5 and ⍺4β7 in CD69+ and CD69- CD4 T cells during the FP of blood. **(B)** Coexpression of CCR5 and ⍺4β7 in CD69+ and CD69- CD4 T cells during the LP of blood. **(C)** Coexpression of CCR5 and ⍺4β7 in CD103+ and CD103- CD4 T cells during the FP of blood. **(D)** Coexpression of CCR5 and ⍺4β7 in CD103+ and CD103- CD4 T cells during the LP of blood. **(E)** Expression of ⍺4β7 in TRM subsets in the EC between FP and LP. **(F)** Coexpression of CCR5 and ⍺4β7 in CD69- CD4 T cells across different phases of EC. **(G)** Coexpression of CCR5 and ⍺4β7 in CD103- CD4 T during the follicular phase (FP) and luteal phase (LP) of the menstrual cycle in the endocervical cells (EC).


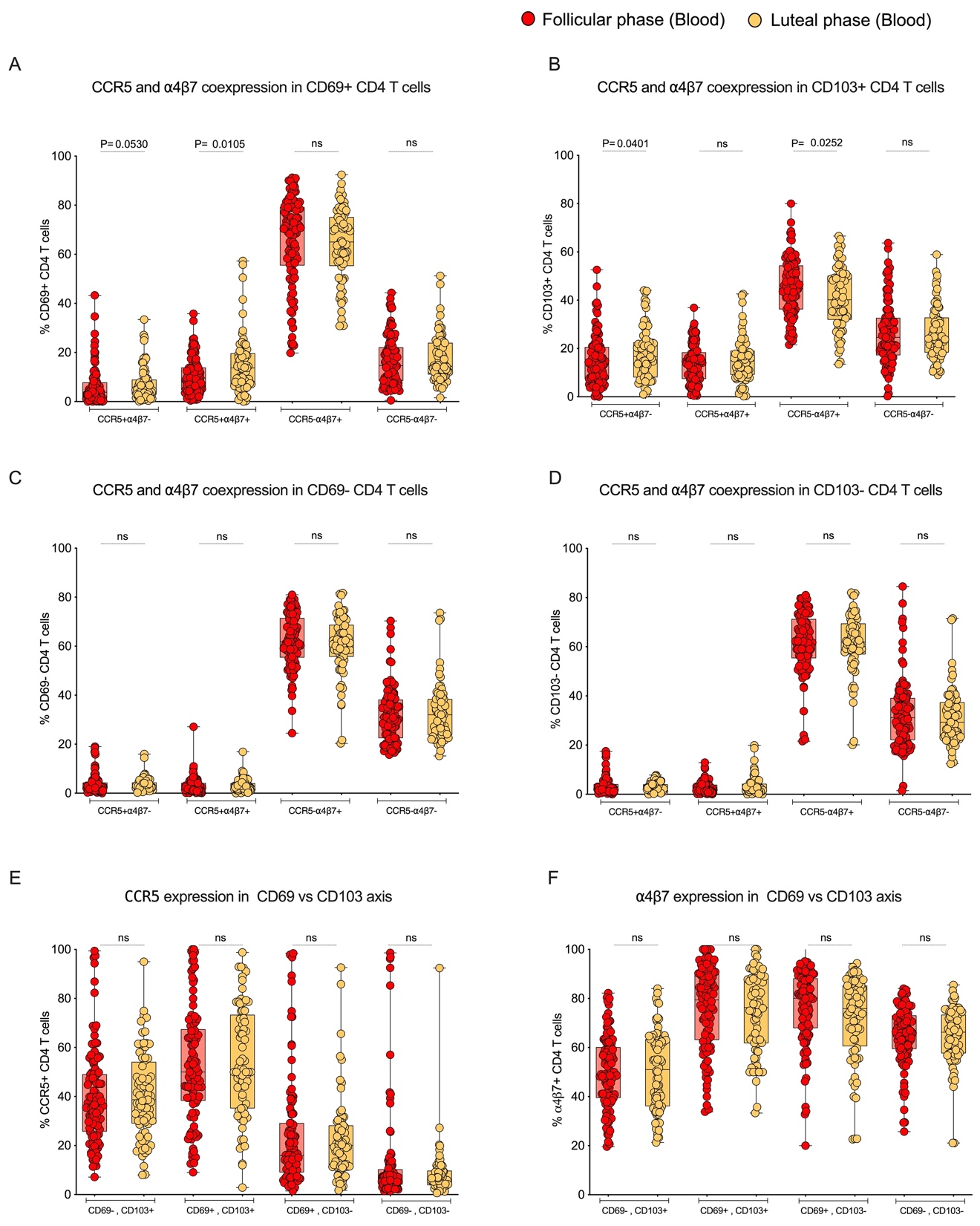


**Supplemental Figure 4. Expression of CCR5 and α4β7 in the in TRM & non-TRM subsets in the endocervix between the follicular and luteal phase of the menstrual cycle:** (**A)** The box plot shows coexpression of CCR5 and ⍺4β7 in CD69+ CD4 T cells during the FP & LP. (**B)** Coexpression of CCR5 and ⍺4β7 in CD103+ CD4 T cells. (**C)** Coexpression of CCR5 and ⍺4β7 in CD69- CD4 T cells. (**D)** Coexpression of CCR5 and ⍺4β7 in CD103- CD4 T cells. (**E)** CCR5 expression in CD69 vs CD103 axis. **(F)** α4β7 expression in CD69 vs CD103 subsets in the blood during the follicular phase (FP) and luteal phase (LP) of the menstrual cycle.

**Supplemental Figure 5. Tissue-resident markers are positively correlated with regulatory T cell and HIV target cells in endocervical samples during the follicular and luteal phase:** (**A)** The plot represents the Spearman correlation between FoxP3 vs CD103+ CD4 T cells in FP. (**B)**. FoxP3 vs CD69+CD103+ CD4 T cells in FP. **(C)**. FoxP3 vs CD69+ CD4 T cells during the FP of endocervical samples. **(D)** Correlation between α4β7 vs CD69+CD103+ CD4 T cells during the luteal phase (LP) of the menstrual cycle in the endocervical samples. **(E)** CCR5 vs CD103+ CD4 T cells in LP. **(F)** α4β7 vs CD103+ CD4 T cells in LP. (**G)** Correlation between MIP-1ß vs CD69+CD103+α4β7+ CD4 T during the luteal phase (LP) of the menstrual cycle in the endocervical samples.

**
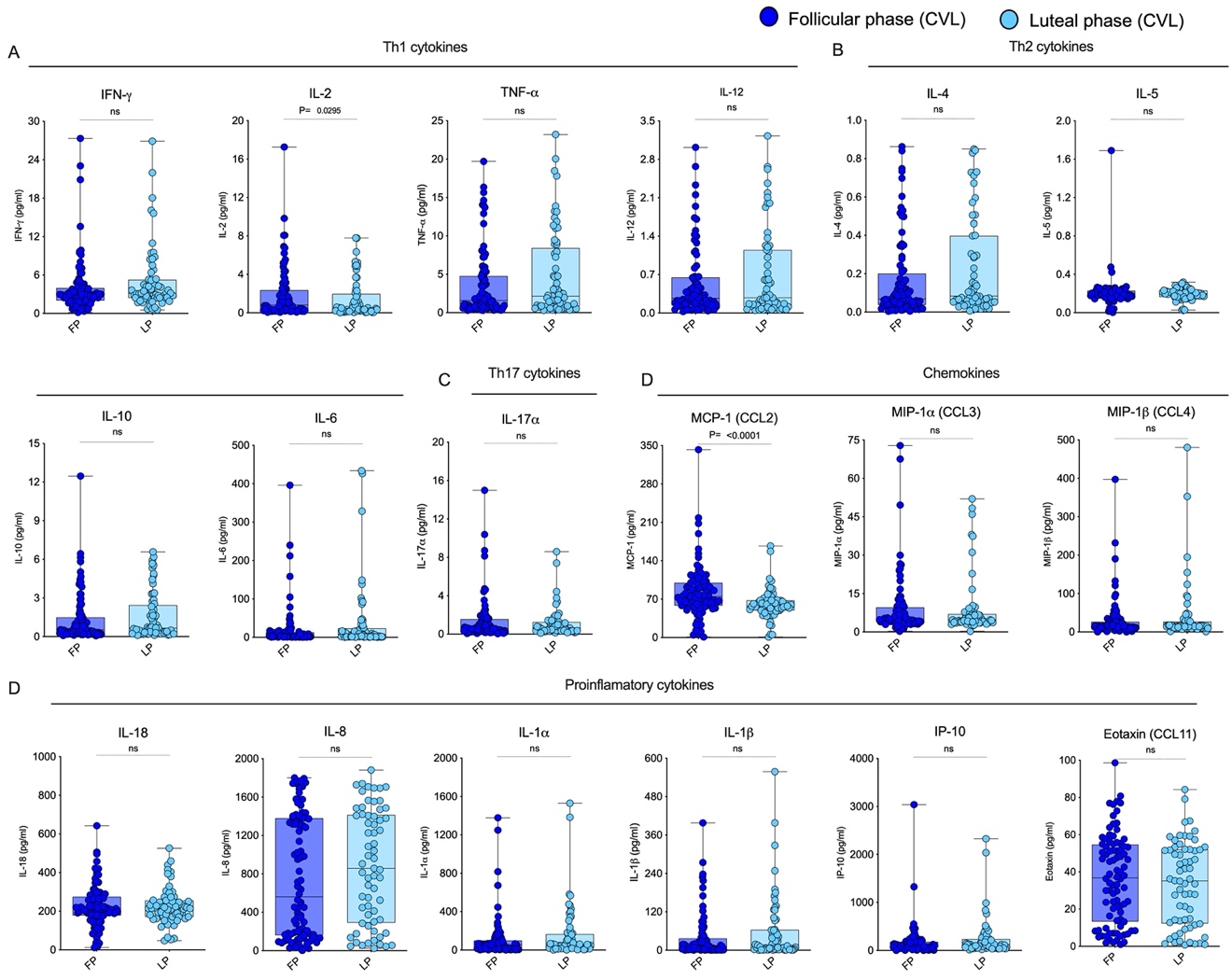
**

**Supplemental Figure 6. Chemokine MCP-1 significantly elevated in the CVL during the follicular phase compared to the luteal phase:** (**A)** Box plot shows Th1 cytokines level. **(B)** Th2 cytokines level. **(C)** Th17 cytokines level. **(D)** Chemokines level. **(E)** Proinflamatory cytokine levels in the cervicovaginal lavage (CVL) during the follicular phase (FP; n=92) and luteal phase (LP; n=67) of the menstrual cycle.

**Supplemental Tables**

**Supplemental Table 1:** Estimated mean cellular marker level within cycle phase in the endocervical samples adjusting for race, and age.

| **Markers** | **Cycle Phase** | **Estimate** | **95% CI LB** | **95% CI UB** | **p-value** |
| --- | --- | --- | --- | --- | --- |
| CD45 | Follicular | 45.45 | 39.26 | 51.64 | 0.6829 |
| CD45 | Luteal | 43.84 | 36.83 | 50.86 |  |
| CD3 | Follicular | 54.16 | 49.59 | 58.73 | 0.9473 |
| CD3 | Luteal | 54.37 | 49.09 | 59.64 |  |
| CD4 | Follicular | 52.91 | 50.56 | 55.26 | 0.1063 |
| CD4 | Luteal | 55.50 | 52.80 | 58.21 |  |
| CCR5 | Follicular | 41.92 | 38.56 | 45.28 | **0.02*** |
| CCR5 | Luteal | 36.57 | 32.71 | 40.44 |  |
| Ki67 | Follicular | 8.80 | 7.38 | 10.22 | **0.03*** |
| Ki67 | Luteal | 6.82 | 5.21 | 8.44 |  |
| FoxP3 | Follicular | 10.81 | 9.24 | 12.38 | **0.0005*** |
| FoxP3 | Luteal | 6.81 | 4.99 | 8.63 |  |
| CD69+CD103+ | Follicular | 8.00 | 5.96 | 10.04 | **0.03*** |
| CD69+CD103+ | Luteal | 5.74 | 3.51 | 7.96 |  |
| CD69 | Follicular | 29.08 | 25.32 | 32.85 | 0.1829 |
| CD69 | Luteal | 25.78 | 21.48 | 30.07 |  |
| HLADR | Follicular | 4.58 | 3.81 | 5.34 | 0.07 |
| HLADR | Luteal | 3.67 | 2.79 | 4.54 |  |
| CD38 | Follicular | 39.71 | 36.59 | 42.82 | 0.3414 |
| CD38 | Luteal | 37.86 | 34.34 | 41.37 |  |
| α4β7 | Follicular | 56.64 | 53.53 | 59.74 | 0.139 |
| α4β7 | Luteal | 59.38 | 55.91 | 62.85 |  |
| α4β7+CCR5+ | Follicular | 24.05 | 21.23 | 26.86 | 0.8188 |
| α4β7+CCR5+ | Luteal | 23.62 | 20.42 | 26.83 |  |

Mixed effect model of cellular marker percentage

The model includes cycle phase (follicular, luteal), race (Asian, Black, White, Else), and age (continuous) in the model. * Indicates significant p <0.05values.

**Supplemental Table 2:** Estimated percentage of HIV Target cells expression level, comparing cycle phase within endocervical samples adjusting for race, age

| **Follicular Vs Luteal** | **Estimate** | **95% CI LB** | **95% CI UB** | **p-value** |
| --- | --- | --- | --- | --- |
| α4β7-CCR5+ | 0.03 | 0.01 | 0.06 | **0.01*** |
| α4β7+CCR5- | -0.07 | -0.10 | -0.03 | **0.0004*** |
| α4β7+CCR5+ | -0.01 | -0.05 | 0.03 | 0.7659 |
| α4β7-CCR5- | 0.01 | -0.02 | 0.04 | 0.5889 |

Linear mixed effect model of α4β7CCR5 percentage

The model includes a combination (α4β7-CCR5+, α4β7+CCR5+, α4β7+CCR5-, and α4β7-CCR5-), cycle phase (follicular, luteal), combo*cycle phase, age (continuous), race (Asian, Black, White, Else).

* Indicates significant p <0.05values.

**Supplemental Table 3:** Estimated mean cellular markers level within cycle phase in the blood adjusting for race, and age.

| **Markers** | **Cycle Phase** | **Estimate** | **95% CI LB** | **95% CI UB** | **p-value** |
| --- | --- | --- | --- | --- | --- |
| CD3 | Follicular | 74.49 | 72.63 | 76.36 | 0.7246 |
| CD3 | Luteal | 74.11 | 72.04 | 76.19 |  |
| CD4 | Follicular | 59.67 | 57.57 | 61.77 | 0.2982 |
| CD4 | Luteal | 60.80 | 58.51 | 63.09 |  |
| CCR5 | Follicular | 7.10 | 6.05 | 8.15 | 0.4231 |
| CCR5 | Luteal | 7.61 | 6.43 | 8.79 |  |
| α4β7 | Follicular | 67.26 | 64.41 | 70.10 | 0.2591 |
| α4β7 | Luteal | 65.48 | 62.35 | 68.62 |  |
| CD38 | Follicular | 60.32 | 57.55 | 63.09 | 0.2274 |
| CD38 | Luteal | 58.30 | 55.20 | 61.41 |  |
| Ki67 | Follicular | 1.92 | 1.73 | 2.11 | 0.8568 |
| Ki67 | Luteal | 1.90 | 1.68 | 2.12 |  |
| FoxP3 | Follicular | 2.53 | 1.99 | 3.06 | 0.9198 |
| FoxP3 | Luteal | 2.50 | 1.91 | 3.08 |  |
| α4β7+CCR5+ | Follicular | 3.59 | 2.85 | 4.33 | 0.1320 |
| α4β7+CCR5+ | Luteal | 4.35 | 3.51 | 5.20 |  |
| α4β7+CCR5- | Follicular | 63.05 | 59.91 | 66.19 | 0.2873 |
| α4β7+CCR5- | Luteal | 61.01 | 57.50 | 64.52 |  |
| α4β7-CCR5+ | Follicular | 3.10 | 2.48 | 3.71 | 0.5240 |
| α4β7-CCR5+ | Luteal | 3.36 | 2.65 | 4.06 |  |

Mixed effect model of cellular marker percentage

The model includes cycle phase (follicular, luteal), race (Asian, Black, White, Else), and age (continuous) in the model.

**Supplemental Table 4:** Estimated percentage of HIV Target cells expression level, comparing cycle phase within the blood, adjusting for race, age

| **Follicular Vs Luteal** | **Estimate** | **95% CI LB** | **95% CI UB** | **p-value** |
| --- | --- | --- | --- | --- |
| α4β7+CCR5+ | -0.01 | -0.03 | 0.00 | 0.0833 |
| α4β7-CCR5+ | -0.01 | -0.02 | 0.00 | 0.2020 |
| α4β7+CCR5- | 0.01 | -0.03 | 0.06 | 0.6286 |
| α4β7-CCR5- | -0.01 | -0.04 | 0.02 | 0.5216 |

Linear mixed effect model of α4β7CCR5 percentage

The model includes a combination (α4β7-CCR5+, α4β7+CCR5+, α4β7+CCR5-, and α4β7-CCR5-), cycle phase (follicular, luteal), combo*cycle phase, age (continuous), race (Asian, Black, White, Else).

**Supplemental Table 5A:** Spearman correlation analysis of target cells and cellular markers in the endocervix during the follicular and luteal phase of menstrual cycle

| **Target cells** | **Cellular marker** | **p-value** | **r-value** |
| --- | --- | --- | --- |
| **Follicular phase** | | | |
| CD4+α4β7-CCR5+ | CD4+Ki67+ | 0.0495 | 0.2137 |
| CD4+α4β7+CCR5+ | CD4+CD69+ | 0.0024 | 0.3777 |
|  | CD4+CD103+ | <0.0001 | 0.3902 |
|  | CD4+CD69-CD103+ | <0.0001 | 0.4279 |
|  | CD4+CD69+CD103- | 0.0087 | 0.2829 |
| **Luteal phase** | | | |
| CD4+α4β7-CCR5+ | CD4+CD69+CD103- | 0.0188 | 0.2975 |
| CD4+α4β7+CCR5+ | CD4+CD103+ | 0.0014 | 0.3976 |
|  | CD4+CD69-CD103+ | 0.0055 | 0.3484 |

**Supplemental Table 5B:** Spearman correlation analysis of target cells and cellular markers in the blood during the follicular and luteal phase of menstrual cycle

| **Target cells** | **Cellular marker** | **p-value** | **r-value** |
| --- | --- | --- | --- |
| **Follicular phase** | | | |
| CD4+α4β7-CCR5+ | CD4+CD103+ | 0.0225 | 0.2473 |
|  | CD4+HLADR+ | 0.0017 | 0.3363 |
|  | CD4+FoxP3+ | 0.0012 | 0.3447 |
|  | CD4+Ki67+ | 0.0088 | 0.2827 |
|  | CD4+CD69-CD103+ | 0.0121 | 0.2712 |
| CD4+α4β7+CCR5+ | CD4+CD103+ | 0.0002 | 0.396 |
|  | CD4+HLADR+ | 0.0055 | 0.2985 |
|  | CD4+Ki67+ | 0.009 | 0.2819 |
|  | CD4+CD69-CD103+ | 0.0012 | 0.3447 |
| CD4+α4β7+CCR5- | CD4+CD38+ | <0.0001 | 0.6014 |
|  | CD4+CD69+CD103- | 0.0158 | 0.2612 |
| **Luteal phase** | | | |
| CD4+α4β7-CCR5+ | CD4+CD103+ | 0.0262 | 0.2823 |
|  | CD4+FoxP3+ | 0.027 | 0.2809 |
|  | CD4+Ki67+ | 0.0405 | 0.2609 |
| CD4+α4β7+CCR5+ | CD4+CD103+ | <0.0001 | 0.4892 |
|  | CD4+FoxP3+ | 0.0328 | 0.2715 |
|  | CD4+HLADR+ | 0.0021 | 0.3827 |
|  | CD4+Ki67+ | 0.0006 | 0.4219 |
|  | CD4+CD69-CD103+ | <0.0001 | 0.5047 |
| CD4+α4β7+CCR5- | CD4+CD38+ | <0.0001 | 0.5144 |

**Supplemental Table 6A:** Spearman correlation analysis of hormones and cellular markers in the endocervix during the follicular and luteal phase of menstrual cycle

| **Hormones** | **Cellular marker** | **p-value** | **r-value** |
| --- | --- | --- | --- |
| **Follicular phase** | | | |
| P4/E2 Ratio | CD4+CCR5+ | 0.0289 | 0.2093 |
|  | CD4+α4β7+CCR5+ | 0.0434 | 0.2005 |
|  | CD4+CD69-CD103+ | 0.0149 | 0.2405 |
|  | CD4+CD69+CD103-CCR5+ | 0.0212 | 0.2216 |
| **Luteal phase** | | | |
| E2 | CD4+CD38+ | 0.0167 | 0.2721 |
|  | CD4+Ki67+ | 0.0117 | 0.286 |
| P4 | CD4+α4β7-CCR5- | 0.0369 | 0.2465 |
| P4/E2 ratio | CD4+α4β7-CCR5- | 0.0056 | 0.3235 |
| FSH | CD4+CD103+ | 0.0498 | 0.2244 |
|  | CD4+CD69+CD103+ | 0.0455 | 0.2365 |
|  | CD69+CD103- | 0.0491 | 0.2328 |

**Supplemental Table 6B:** Spearman correlation analysis of hormones and cellular markers in the blood during the follicular and luteal phase of menstrual cycle

| **Hormones** | **Cellular marker** | **p-value** | **r-value** |
| --- | --- | --- | --- |
| **Luteal phase** | | | |
| P4 | CD4+CD69+CD103- | 0.0229 | 0.2678 |
| P4/E2 Ratio | CD4+CD69+CD103- | 0.0267 | 0.2612 |

**Supplemental Table 6C:** Spearman correlation analysis of plasma hormones and CVL cytokines during the follicular and luteal phase of menstrual cycle

| **Hormones** | **Cytokines** | **p-value** | **r-value** |
| --- | --- | --- | --- |
| **Follicular phase** | | | |
| E2 | IL-2 | 0.0078 | 0.2852 |
|  | MCP-1 | 0.0387 | 0.2159 |
| LH | MCP-1 | 0.0473 | 0.2074 |
| **Luteal phase** | | | |
| P4/E2 ratio | IL-1α | 0.004 | 0.3521 |

**Supplemental Table 7A:** Spearman correlation analysis of cytokines and cellular markers in the endocervix during the follicular and luteal phase of menstrual cycle

| **Cytokines** | **Cellular marker** | **p-value** | **r-value** |
| --- | --- | --- | --- |
| **Follicular phase** | | | |
| IFN-γ | CD4+FoxP3+ | 0.0072 | 0.2782 |
|  | CD4+Ki67+ | 0.0027 | 0.309 |
| IL-2 | CD4+FoxP3+ | 0.0163 | 0.2645 |
|  | CD4+Ki67+ | 0.011 | 0.2795 |
| IL-4 | CD4+FoxP3+ | 0.0172 | 0.2534 |
| IL-5 | CD4+CD69-CD103+CCR5+ | 0.0377 | 0.2259 |
| IL-6 | CD4+CD69-CD103+CCR5+ | 0.0243 | 0.236 |
| MCP-1 | CD4+CD69+CD103- | 0.0127 | 0.2691 |
| Eotaxin | CD4+Ki67+ | 0.005 | 0.297 |
| **Luteal phase** | | | |
| IL-17 | CD4+α4β7+CCR5+ | 0.0459 | 0.2609 |
| IL-18 | CD4+CD69+ | 0.0189 | 0.2904 |
| MIP-1β | CD4+CD69+CD103+α4β7+ | 0.0434 | 0.2534 |

**Supplemental Table 7B:** Spearman correlation analysis of cytokines and cellular markers in the blood during the follicular and luteal phase of menstrual cycle

| **Cytokines** | **Cellular marker** | **p-value** | **r-value** |
| --- | --- | --- | --- |
| **Follicular phase** | | | |
| IFN-γ | CD4+Ki67+ | 0.0432 | 0.2112 |
|  | CD4+CD69+CD103+ | 0.0452 | 0.2178 |
|  | CD4+CD69+CD103- | 0.0416 | 0.2215 |
| IL-12 | CD4+CD69+CD103+ | 0.0257 | 0.2558 |
| IL-17α | CD4+CD38+ | 0.0225 | 0.2403 |
|  | CD4+CD169+CD103- | 0.0075 | 0.2915 |
| IL-18 | CD4+CD69+ | 0.024 | 0.2352 |
|  | CD4+α4β7+CCR5+ | 0.0415 | 0.2216 |
| IL-8 | CD4+CD169+CD103- | 0.0452 | 0.2179 |
| IL-1α | CD4+FoxP3+ | 0.0003 | 0.3739 |
|  | CD4+HLADR+ | 0.0312 | 0.226 |
| **Luteal phase** | | | |
| IFN-γ | CD4+α4β7+CCR5- | 0.0414 | 0.2641 |
| TNF-α | CD4+α4β7+ | 0.0215 | 0.2847 |
|  | CD4+α4β7+CCR5- | 0.0394 | 0.2667 |
| IL-6 | CD4+α4β7+ | 0.0214 | 0.285 |
|  | CD4+CD38+ | 0.0306 | 0.2685 |
|  | CD4+α4β7+CCR5- | 0.0042 | 0.3646 |
| IL-17α | CD4+α4β7+CCR5- | 0.039 | 0.2695 |
| IL-1β | CD4+α4β7+ | 0.0277 | 0.2731 |
|  | CD4+α4β7+CCR5- | 0.0422 | 0.2631 |
| IP-10 | CD4+α4β7+CCR5- | 0.0091 | 0.3339 |
| Eotaxin | CD4+α4β7+CCR5- | 0.0006 | 0.4344 |
